# Letter in Response to the Article “Glenosphere Size Does Not Matter in Reverse Total Shoulder Arthroplasty”

**DOI:** 10.1055/s-0044-1800940

**Published:** 2025-04-11

**Authors:** Akshar V. Patel, Christopher A. White, Troy Li, Bradford O. Parsons, Evan L. Flatow, Paul J. Cagle

**Affiliations:** 1 Departamento de Cirurgia Ortopédica, Icahn School of Medicine at Mount Sinai, Nova Iorque, NY, Estados Unidos

To the Editor,

My co-authors and I thank Dr. Bonadiman for taking the time to respond to our manuscript, “Glenosphere Size Does Not Matter in Reverse Total Shoulder Arthroplasty.” 1 We have prepared a reply addressing the comments he has raised.

The work in question represents a single-surgeon, single-implant study. The capacity to have a single implant and single surgeon removes many variables, and we believe this is a great strength of the dataset. All patients in the study received a Trabecular Metal Reverse Shoulder System (Zimmer Biomet, Warsaw, IN, United States). Additionally, the subscapularis was repaired in all of our patients. The choice to repair was a preference of the operative surgeon. This choice predated many of the published papers demonstrating the impact of choosing to repair or to not repair the subscapularis. As such, a protocol for why to repair was not possible.

Regarding the postoperative protocol, the study was not a randomized controlled trial. As such, a standardized methodology of postoperative treatment was not established a priori. As a rule, all patients were asked to wear a sling for six weeks, and active motion did not begin until the sling was removed.

At the time this paper was written, our institutional shoulder arthroplasty registry included patients from 1987 to 2019 who had undergone hemiarthroplasty or anatomic or reverse TSA. However, the patients in the study had undergone a reverse TSA between 2005 and 2014. This is illustrated in the reported follow-up periods.

The postoperative measurements were reported until the final follow-up visit. Each patient had a minimum of 2.0 years of follow-up. The mean follow-up in each group was of 6.7 ± 3.3 years for the 36-mm cohort and of 4.8 ± 3.1 years for the 40-mm cohort.

Similarly, all patients in the study underwent reverse TSA after the invention of the Simple Shoulder Test in 1993. Thus, the test was applied to all patients pre- and postoperatively.

We thank the journal for publishing our manuscript and for this opportunity to comment on it in further detail.
