# Supplementary material for: Letter in Response to the Article “Glenosphere Size Does Not Matter in Reverse Total Shoulder Arthroplasty”
Source: Rev Bras Ortop (Sao Paulo). 2025 Apr 11;60(1):s00441800940. doi: 10.1055/s-0044-1800940 (PMC12020542; doi:10.1055/s-0044-1800940)
Supplement: Related texts

# Research article: Carta em resposta ao artigo “O tamanho da glenosfera não importa na artroplastia total reversa de ombro”

Ao Editor,

Meus coautores e eu agradecemos ao Dr. Bonadiman por responder ao nosso manuscrito “O tamanho da glenosfera não importa na artroplastia total reversa de ombro”. 1 Preparamos uma resposta abordando os comentários levantados por ele.

O trabalho em questão representa um estudo de implante único feito por um só cirurgião. A capacidade de ter um só implante e um só cirurgião remove muitas variáveis, e acreditamos que esta é uma grande força dos dados. Todos os pacientes no estudo receberam um Sistema de Ombro Reverso Trabecular Metal (metal trabeculado; Zimmer Biomet, Warsaw, IN, Estados Unidos). Além disso, todos os nossos pacientes foram submetidos ao reparo do subescapular. A escolha do reparo foi uma preferência do cirurgião, e antecedeu muitos dos artigos publicados que demonstram o impacto da escolha de reparar ou não reparar o subescapular. Como tal, um protocolo sobre o motivo do reparo não foi possível.

Em relação ao protocolo pós-operatório, o estudo não foi um ensaio clínico randomizado controlado; assim, uma metodologia padronizada de tratamento pós-operatório não foi estabelecida previamente. Como regra, solicitou-se a todos os pacientes que usassem uma tipoia por seis semanas, e a movimentação ativa não começou até a remoção da tipoia.

Quando o artigo foi escrito, nosso cadastro institucional de artroplastia de ombro (ATO) incluía pacientes de 1987 a 2019 submetidos a hemiartroplastias ou a ATO anatômica ou reversa. No entanto, os pacientes do estudo tinham sido submetidos uma ATO reversa entre 2005 e 2014. Isso é ilustrado nos tempos de acompanhamento relatados.

As medidas pós-operatórias foram relatadas até a última consulta de acompanhamento. Cada paciente foi acompanhado por, no mínimo, 2,0 anos. A média do acompanhamento foi de 6,7 ± 3,3 anos na coorte de 36 mm, e de 4,8 ± 3,1 anos na coorte de 40 mm.

Da mesma forma, todos os pacientes no estudo foram submetidos a ATO reversa após a invenção do Simple Shoulder Simple Test (“Teste Simples de Ombro”) em 1993. Assim, todos os pacientes foram submetidos ao teste antes e depois da cirurgia.

Agradecemos ao periódico pela publicação de nosso manuscrito e por esta oportunidade de comentá-lo em mais detalhes.
